# Increase in bloodstream infections caused by *emm*1 group A *Streptococcus* correlates with emergence of toxigenic M1_UK_, Belgium, May 2022 to August 2023

**DOI:** 10.2807/1560-7917.ES.2023.28.36.2300422

**Published:** 2023-09-07

**Authors:** Juan Pablo Rodriguez-Ruiz, Qiang Lin, Christine Lammens, Pierre R Smeesters, Stefanie van Kleef-van Koeveringe, Veerle Matheeussen, Surbhi Malhotra-Kumar

**Affiliations:** 1 Laboratory of Medical Microbiology, Vaccine and Infectious Disease Institute, University of Antwerp, Wilrijk, Antwerp, Belgium; 2 National Reference Centre for invasive β-haemolytic streptococci, Belgium; 3 Laboratory of Molecular Bacteriology, Université Libre de Bruxelles (ULB), Brussels, Belgium; 4 Department of Paediatrics, Brussels University Hospital, Academic Children Hospital Queen Fabiola, Université Libre de Bruxelles (ULB), Brussels, Belgium; 5 Laboratory of Microbiology, University Hospital Antwerp, Edegem, Antwerp, Belgium

**Keywords:** Streptococcus pyogenes, emm1, invasive group A Streptococcus, bloodstream infections

## Abstract

Many European countries have recently reported upsurges in invasive group A *Streptococcus* (iGAS) infections, mainly caused by *emm*1 *Streptococcus pyogenes*, specifically the toxigenic M1_UK_ lineage. We present the epidemiology of *emm*1 causing iGAS in Belgium during 2018–August 2023, and describe an emergence of the toxigenic M1_UK_ lineage in Belgium in mid-2022 that was observed as an increase in bloodstream infections caused by *emm*1 *S. pyogenes* that continued into 2023.

Since mid-2022, invasive group A *Streptococcus* (iGAS) infections caused by *Streptococcus pyogenes* harbouring *emm*1, which encodes the M1 protein, have been increasingly reported across different European countries [1-4]. Specifically, this increase seems to be related to an increase in the proportion of the toxigenic M1_UK_ lineage [5] compared to the original M1_global_ lineage [6,7]. The M1_UK_ lineage differs from the original by 27 defining single nucleotide polymorphisms (SNPs), which leads to an increased expression of the superantigen gene *speA* [8]. Since June 2023, another M1 sublineage was reported from Denmark (M1_DK_), which is characterised by the presence of *speC* and 15 defining SNPs [4]. In Belgium, a remarkable increase in iGAS infections was also observed from mid-2022, mostly caused by *emm*1 *S. pyogenes*. Here, we present the epidemiology, genetic characteristics and associated lineages of *emm*1 *S. pyogenes* causing iGAS infections in Belgium.

## Invasive group A *Streptococcus* in Belgium in 2022–2023

**Figure 1:**
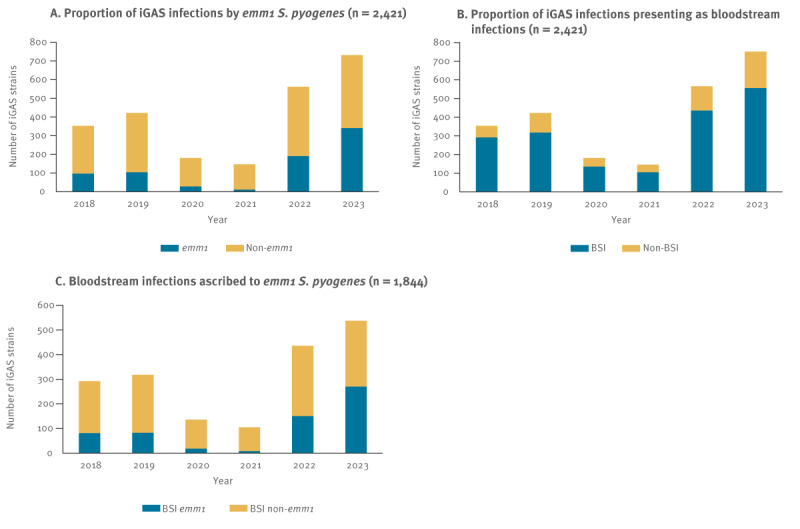
Overview of invasive group A *Streptococcus* infections caused by *Streptococcus pyogenes*, Belgium, 2018–August 2023

BSI: bloodstream infections; iGAS: invasive group A *Streptococcus*; *S. pyogenes: Streptococcus pyogenes*.

**Figure 2:**
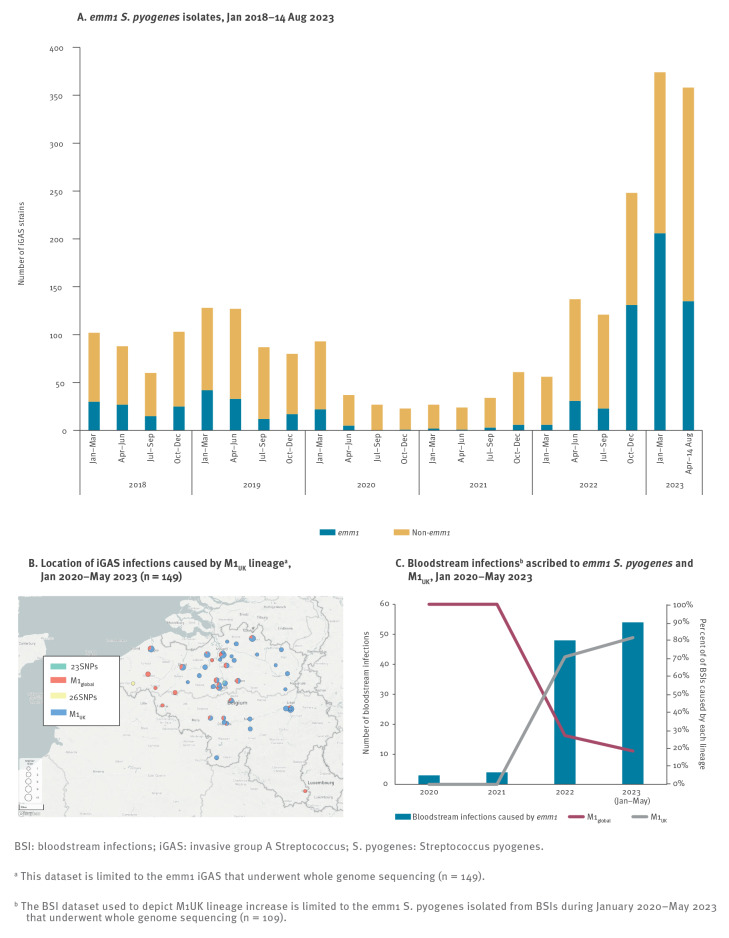
Distribution of *emm*1 invasive group A *Streptococcus* and bloodstream infections caused by the M1_UK_ lineage, Belgium, January 2018–August 2023

BSI: bloodstream infections; iGAS: invasive group A *Streptococcus*; *S. pyogenes: Streptococcus pyogenes*.

^a^ This dataset is limited to the *emm*1 iGAS that underwent whole genome sequencing (n = 149).

^b^ The BSI dataset used to depict M1_UK_ lineage increase is limited to the *emm*1 *S. pyogenes* isolated from BSIs during January 2020–May 2023 that underwent whole genome sequencing (n = 109).

In Belgium, the total number of iGAS infections increased 1.3 to 1.6-fold in 2022 and 1.8- to 2-fold in 2023 compared to the pre-COVID-19 years (2018 and 2019, respectively) (Figure 1A). Of all iGAS infections, 45% (341/752) and 34% (191/566) were caused by *emm*1 *S. pyogenes* in 2023 (data available up to 14 August 2023) and in 2022, respectively, as compared to 25−27% in 2018 (97/354) and 2019 (104/422) (p ≤ 0.0056, Fisher’s exact test, Figure 1A and 2A). Although *emm*1 has been the predominant *emm* type among Belgian iGAS, *emm*1 comprises only 10%−25% of the yearly iGAS strains since the start of the surveillance in 2012 [9]. Age-based stratification of *emm*1 iGAS infections from May 2022 up to 14 August 2023 did not show a predilection for any age group (Table). Of the 518 *emm*1 iGAS infections reported during this period, 31% (n = 160) were identified in the 0–12 age group, 16% (n = 85) in the 18–40, and 25% and 26% in the 41–65 (n = 129) and 65 and older (n = 135) age groups, respectively (Table).

**Table:** Number of *emm*1 invasive group A *Streptococcus* (iGAS) infections, 1 May 2022–14 August 2023 (n = 518), and number of M1_UK_ lineage among sequenced *emm*1* S. pyogenes* causing iGAS infections, May 2022–May 2023 (n = 130) by age, Belgium

Case characteristics						*emm*1 iGAS infections^a^				Sequenced *emm*1 iGAS^b^		M1**_UK_** in iGAS infections^d^		M1**_global_** in iGAS infections^d^		M1**_UK_** causing BSIs^e^	
Age range (years)	Total	Sex^b,c^				May−Dec 2022		Jan−Aug 2023									
		Males		Females													
		n	%	n	%	n	%	n	%	n	%	n	%	n	%	n	%
0–12	160	86	54	74	46	64	36	96	28	40	25	27	68	12	30	19	70
13–17	4	2	50	2	50	1	1	3	1	1	25	1	100	0	0	1	100
18–40	85	36	42	49	58	26	15	59	17	34	40	26	76	8	24	19	73
41–65	129	76	59	53	41	46	26	83	24	31	24	27	87	4	13	22	81
> 65	135	65	48	69	51	38	21	97	28	22	16	20	91	2	9	16	80
Unknown	5	3	60	2	40	2	1	3	1	2	40	1	50	1	50	1	100
Total	518	268	52	249	48	177	100	341	100	130	25	102	78	27	21	78	76

BSIs: bloodstream infections; iGAS: invasive group A *Streptococcus*.

^a^ For *emm*1 iGAS infections: % based on total isolates in that period.

^b^ For sex and sequenced isolates: % based on total number of isolates per age group.

^c^ Sex is unknown for one case in the > 65 age group.

^d^ For M1 isolates: % based on sequenced isolates. The dataset for lineage assignment is limited to the *emm*1 iGAS isolated during May 2022–May 2023 that underwent whole genome sequencing (n = 130).

^e^ For M1_UK_ causing BSI: % based on M1_UK_ causing iGAS.

The ratio of bloodstream infections (BSI) and non-BSI remained fairly constant, with BSI comprising 74−82% of the iGAS infections from 2018−23 (Figure 1B). However, the proportion of BSIs caused by *emm*1 *S. pyogenes*, which was 26−28% in 2018−19, dropped to 9%−14% during the COVID-19 pandemic (2020–21). In 2022, the proportion increased to 35%, and in 2023 constituted almost 50% of all BSIs in Belgium (p ≤ 0.0105 when comparing 2023 with the other years, Fisher’s exact test, Figure 1C). Patients with BSIs included those where *emm*1 *S. pyogenes* was isolated from blood cultures, or those with a primary differential diagnosis of septicaemia or meningitis, with or without other conditions, where *emm*1 *S. pyogenes* was isolated from another sample type, e.g. cerebrospinal fluid, pleural fluid.

## Whole genome sequencing

An at-random selection of *S. pyogenes* isolated from patients with suspected iGAS infections, which were submitted to the National Reference Centre for invasive β-haemolytic streptococci between January 2020 and May 2023 and typed as *emm*1, were subjected to whole genome sequencing (n = 149, llumina, MiSeq). Approximately, 20–45% of all Belgian *emm*1 iGAS strains between January 2020 and May 2023 were sequenced (2020: 9/28, 2021: 5/11, 2022: 66/191 and 2023: 69/341. Strains were isolated from blood (90/149, 60%), wounds (13/149, 9%), pleural fluid (12/149, 8%), biopsies (8/149, 5%) and other invasive sample types (26/149, 17%) with a clinical presentation of septicaemia, streptococcal toxic shock syndrome (STSS), erysipelas, cellulitis, necrotising fasciitis, necrotising pneumonia, empyema, osteomyelitis and meningitis.

After initial quality assessment and trimming with FastQC v0.11.9 (https://www.bioinformatics.babraham.ac.uk/projects/fastqc) and TrimGalore v0.6.7 (https://wiki.rc.usf.edu/index.php/TrimGalore), a whole genome alignment against the *emm*1 reference MGAS5005 was generated with Snippy v4.6.0 (https://github.com/tseemann/snippy), and the SNP distance matrix was extracted from the whole genome alignment using snp-dists v0.7.0 (https://github.com/tseemann/snp-dists). Isolates were assigned to the M1_UK_ or the M1_DK_ lineages based on the presence of the 27 or 15 defining SNPs in the whole genome alignment, as described previously [4,5]. Genome assemblies were generated with SPAdes v3.15.0 [10] and were annotated with prokka v1.14.5 [11]. Annotated assemblies were further analysed with BacPipe [12] for detection of acquired antimicrobial resistance genes based on the CARD database. Metadata was visualised using Microreact online interface. 

Remarkably, BSI isolates constituted 73% (109/149) of the sequenced *emm*1 *S. pyogenes* in this study. The *emm*1-associated BSIs were detected in patients of all ages, ranging from 0−92 years. Sequencing identified the rapid increase in the proportion of the M1_UK_ lineage among iGAS isolates in Belgium during 2022–23 (Figure 2B and C). This toxigenic lineage was already detected in Belgium in one BSI sample as early as January 2020. No isolate was identified that presented the 15 SNPs defining the recently described M1_DK_ lineage, although six (two M1_global_ and four M1_UK_) presented the *speC* superantigen gene. From May 2022 to May 2023, of 130 *emm*1 isolates sequenced, 102 (78%) were M1_UK_, and one presented 23 of the 27 defining SNPs, making M1_UK_ the currently dominant *emm*1 lineage in Belgium (Figure 2B and C). This lineage also constituted 72% (78/109) of the BSI-associated *emm*1 isolates sequenced in this study. However, disease outcomes did not vary notably between patients developing infections caused by M1_UK_ or M1_global_. Of the 13 of 149 patients who died, eight were infected with M1_UK_ and were aged 7, 40, 56, 65 and over 65 (n = 4) years. Five patients who died were infected with M1_global_ (ages 0, 1 (n = 2), 33 and 34 years). Of these 13 patients, twelve had been diagnosed with BSI (septicaemia, with or without pneumonia, fasciitis or STSS), while one presented with pneumonia. In our dataset, neither severity of disease nor disease presentation varied between the two lineages. 

**Figure 3:**
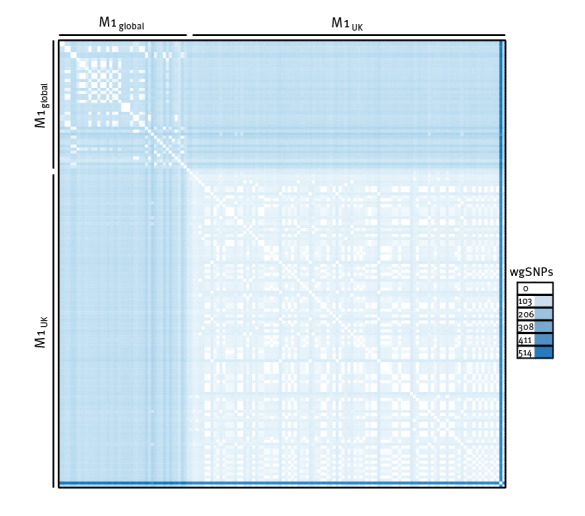
Single nucleotide polymorphism (SNP) distance matrix representing number of whole genome SNPs between all included isolates, Belgium, Jan 2020–May 2023 (n = 149)

Within the M1_UK_ isolates studied here, we identified on average 41 whole genome SNPs, indicating higher genome stability, in comparison to the M1_global_ isolates, which were isolated during the same timeframe, and presented on average 85 whole genome SNPs within the lineage (Figure 3). However, one M1_UK_ isolate presented a remarkably higher number of SNPs compared to the rest of isolates (452 whole genome SNPs on average), despite showing the same *emm* type and ST, and will require further investigation. Remarkably, M1_UK_ isolates showed on average 105 whole genome SNPs when compared to the M1_global_ isolates. 

wgSNPs: whole genome single nucleotide polymorphisms.

Most of the isolates (139/149, 93%) belonged to sequence type (ST)28, one M1_global_ isolate was ST785 and seven M1_UK_ isolates were ST1357. Screening for antimicrobial resistance genes showed that *mefE*, which encodes a macrolide resistance-conferring efflux pump, was present in eight isolates (seven M1_UK_ and one M1_global_), tetracycline resistance-encoding genes *tetU* and *tetM* were identified in four isolates (three M1_UK_ and one M1_global_), and one isolate (M1_UK_) presented with the aminoglycoside resistance determinant, *aad* [6]. Although prevalence of antimicrobial resistance genes in the M1_UK_ lineage was rather low, this was higher than among M1_global_ isolates. The most commonly observed gene was *mefE*, as also reported in the original description of the M1_UK_ lineage [5].

## Discussion

The increase in *emm*1 iGAS observed during mid-2022 until August 2023 in Belgium was primarily because of an upsurge in BSIs caused by *emm*1 *S. pyogenes*. Remarkably, this coincided with the lifting of mandatory use of protective face masks in Belgium. Other countries have observed a similar increase in *emm*1 iGAS infections with different clinical presentations after the removal of COVID-19-related non-pharmaceutical protective measures. A rise in pleural empyema was reported in Scotland and England [1,13], meningitis in the Netherlands [6], and general infections in the Netherlands [2], France [3], Denmark [4] and England [7]. 

Our observations were similar to previous studies reporting that the M1_UK_ lineage does not lead to more severe infections, although the *emm*1 genotype itself has been linked to higher virulence and requirement for intensive care [3,4]. In Belgium, the *emm*1-associated BSIs or those caused by the M1_UK_ lineage did not show a proclivity for any age group, in contrast to previous findings that M1_UK_ infections are more common among the paediatric population [7]. The success of the M1_UK_ lineage has been hypothesised to be derived from the accumulation of SNPs in the genome that provide a fitness advantage in colonising the host [8], which caused this lineage to become predominant before the pandemic in the United Kingdom [5]. Genome stability, measured by accumulation of new SNPs in a defined timeframe, was higher among the M1_UK_ isolates than the M1_global_ studied here. These data support the hypothesis that the SNPs accumulated by the M1_UK_ lineage are sufficient to provide a fitness advantage, which – coupled with a potentially lowered herd immunity to *S. pyogenes* because of decreased exposure during the pandemic and the increase in other respiratory viral infections – might explain the upsurge in iGAS infections reported in the 2022/23 winter season in many European countries.

Our investigation had some limitations. We studied a selection of the *emm*1 *S. pyogenes* that were all isolated from invasive infections, which did not allow a contextual analysis of other prevalent genotypes or of the prevalence of M1_UK_ among non-invasive GAS infections. Despite the limitations, analysis of *emm*1 iGAS from years both pre- and post-COVID-19 pandemic nonetheless facilitated a clear picture of *emm*1 iGAS dynamics in Belgium.

## Conclusion

The toxigenic M1_UK_ lineage emerged in Belgium in mid-2022 and was observed as an increase in bloodstream infections caused by *emm*1 *S. pyogenes* that has continued until August 2023. These data call for increased vigilance and a sustained real-time monitoring of iGAS infections in Europe.
